# Naming abilities: Differentiation between objects and verbs in aphasia

**DOI:** 10.1590/S1980-57642010DN40400006

**Published:** 2010

**Authors:** Luisa Carmen Spezzano, Márcia Radanovic

**Affiliations:** 1 Speech Pathologist, Post-graduate student, Department of Neurology, University of São Paulo School of Medicine.; 2 MD, MsC, PhD, Department of Neurology, University of São Paulo School of Medicine.

**Keywords:** aphasia, language, names

## Abstract

Cognitive Neuropsychology aims to understand the processing mechanisms of normal and injured brain, by means of functional architectural models of information processing. Naming is one of the most important abilities in linguistic processing. Naming of different semantic and grammatical categories differ in their lexical properties and have distinct neuroanatomical substrates. We reviewed literature data on the differences between nouns and verbs in aphasic subjects reported by scientific publications in the form of indexed articles. Studies on naming abilities tended to emphasize the differentiation between nouns and verbs both in their lexical properties and neuroanatomical substrates. Functional neuroimaging studies have improved the state of knowledge regarding category-specific naming abilities, but further studies on different types of aphasia and the use of naming abilities in different contexts are warranted.

## Abstract (pt)

A Neuropsicologia Cognitiva busca compreender o funcionamento cerebral através de modelos de arquiteturas funcionais do processamento da informação. A nomeação constitui uma das tarefas mais importantes no processamento da linguagem. A nomeação de diferentes categorias semânticas e gramaticais difere em suas propriedades lexicais e possui substratos neuroanatômicos distintos. Revisamos dados da literatura sobre a diferenciação da habilidade de nomeação de categorias específcas e seus substratos neurais em afásicos, por meio de publicações científicas em forma de artigos indexados. Estudos sobre as habilidades de nomeação enfatizam a distinção entre substantivos e verbos, em suas propriedades lexicais e substratos neuroanatômicos. Pesquisas com exames de neuroimagem funcional tem contribuído para o avanço do conhecimento da nomeação, porém ainda são necessários estudos que relacionem diferentes categorias semânticas nas diferentes afasias e contextos lingüísticos.

Research lines with different theoretical approaches and based on different models, such as the anatomoclinical, cognitivism, and psychosocial methods have emerged and seek to better understand the alterations in communication that result from neurological disorders in adults.^1^

Cognitive Neuropsychology aims to understand the processing mechanisms of normal and injured brain by means of functional architectural models of information processing. It emphasizes that linguistic abilities are organized into multiple processes within subsystems that interact with each other, while maintaining some degree of independency.^2^

Figure 1 shows how mental representations are influenced by functional architecture and their transformations. This example depicts lexical processing and how different abilities (naming, reading, and writing) interact with each other through neural networks.^2^

**Figure 1:**
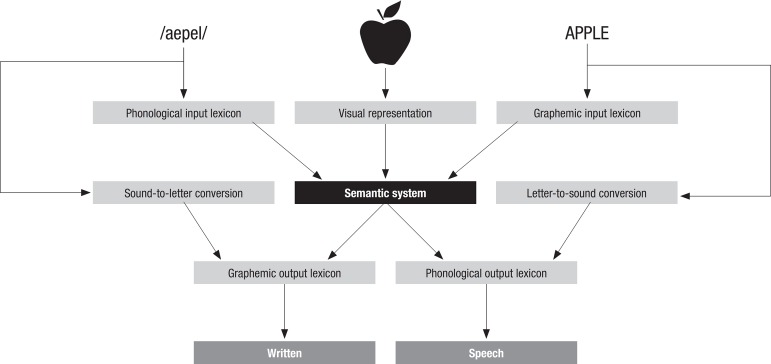
Representation of components involved in lexical processing (cited in Hillis, 20082).

Naming is one of the most important abilities in linguistic processing. The task requires retrieval of phonological and semantic information, which is organized in a memory system and assessed depending on the specificities of a given stimulus.^3,4,5^

Based on the principles of Cognitive Neuropsychology, the visual confrontation naming process (in which the subject has to name an object or representational picture based on visual input) comprises three stages:

[1] Identification of the represented object, which activates its mental structural representation;
[2] Access to its semantic representation, which allows the object to be recognized, and
[3] Lexicalization, or activation of its phonological representation, by which the name of the picture or object is retrieved and uttered.^2,4,5^

Naming involves lexical and non-lexical processing. The first refers to the storage and retrieval of semantic information and abstract representations connected with a particular word. The second refers to the detection and perception of the visual stimuli that triggers the lexical process.

Spezzano^6^ have showed that the higher the schooling, the greater the familiarity of subjects with the objects, which become more visually concordant and perceptually simple. Consequently, the subjects tend to make fewer naming errors, and to display less latency in their responses.

Naming disturbances comprise paraphasias (or substitutions), which may be phonemic (substitution of one phoneme for another), semantic (substitution of one word for another semantically-related word, as in “*boss*” for “*president*”, verbal (a combination of the former), neologisms (the creation of non words), circumlocutions (an attempt by the subject to “explain” the characteristics of items they cannot name properly), and perseverations (repetition of words or fragments of sentences, which are sometimes meaningless)^7^. The causes of this disorder include vascular etiologies (such as stroke), brain trauma, inflammatory processes and tumors.^8^

Among the language disturbances observed, aphasia is the most frequent, defined as a linguistic impairment caused by a neurological lesion that may compromise comprehension and/or production of language in its oral or written forms.^9^

The need for cues in naming tasks can be controlled and indicates specific difficulties according to their nature. The need for phonemic cues, whereby the first phoneme or syllable of the word is given to the subject by the examiner, is found in lexical access difficulties. The necessity of semantic cues, in which the meaning of the word (through its function, for example), indicates a visual deficit, or inability to recognize the picture or object.

The basic neurofunctional model of naming^4^ explains the different alterations in these abilities, as shown in Figure 2.

**Figure 2:**
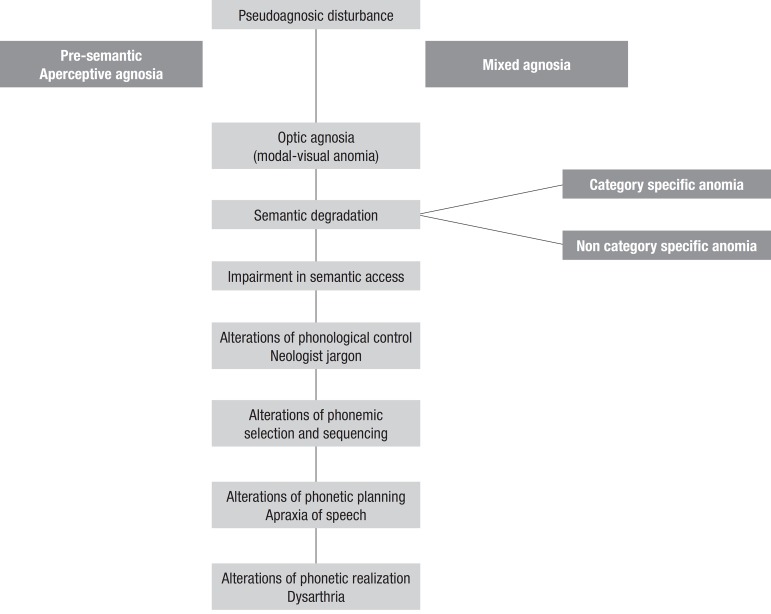
Alterations in naming abilities.

Studies derived from injured brains have raised discussion over differences between verb and noun naming. According to Campos^10^ and Martins^11^ naming of words of different semantic and grammatical categories differ in their lexical properties and have distinct neuroanatomical substrates.

Naming abilities are evaluated by means of tests of general denomination, such as the Snodgrass and Vanderwart pictures,^3^ and the Boston Naming Test,^12,13^ or through specific denomination tests, which assess different semantic and grammatical categories such as the Specific Categories Naming Test^14^ and the Verb and Objects Naming Battery.^15^

The aim of this study was to review literature data on the differences between nouns and verbs in aphasic subjects, and their neural substrates.

The research publications reviewed here were retrieved by computer search of the main databases of scientific Neuropsychology and Cognitive Neurology oriented periodicals (MEDLINE, SCOPUS, ELSEVIER, PSYCHINFO), using the following key-words:

[1] Aphasia;
[2] Naming;
[3] Category-specific naming, and
[4] Neural substrates for verb and object naming.

As inclusion criteria, the selected papers had to have been published in English or Portuguese, within the last ten years (2000 to 2010). Textbooks were also consulted in order to enhance the theoretical aspects of the Discussion.

The results were divided into 1. Double-dissociation in noun and verb naming; 2. Neural substrate of the linguistic processing of nouns and verbs; and 3. Naming processes in aphasia.

The search based on the key-words outlined above yielded 54 papers, of which 13 referred to naming alterations in developmental language disturbances in children. Of the 41 papers related to naming processing in adults, 17 referred to naming disturbances in dementia. Therefore, 24 papers were selected for analysis in this review.

## Double dissociation between nouns and verbs

Levelt^16^ proposed a linguistic theory on word production, which encompasses the following levels: conceptual elaboration, lexical selection (lemma), morphological encoding (morpheme), phonological and phonetic encoding (phoneme and phonetic elaboration), and articulation of speech.

From this linguistic theory perspective, cognitive researchers have suggested differentiation between verbs and nouns in lexical access (lemma selection) due to diverse semantic and grammatical characteristics.^17,18^

Nouns and verbs are different in that nouns refer to the names of objects and have an argument function, while verbs express actions and have a predicative function.^17,19-23^

Considering that nouns represent names of objects, in Cognitive Neuropsychology studies, their concepts may be superimposed and definitions may become similar. Nouns have two notions: comprehension (recognition of images from a spoken or written stimulus) and extension (number of subjects to whom the meaning of the word refers).

Nouns vary according to gender, number and grade. They contain specific semantic features, such as being concrete or abstract, living and non living (innate), human or non human, as well as possessing characteristics regarding place and time.^21^

Martins^11^ reported that aphasic subjects with left cerebral lesion performed worse in naming non living objects. However, it must be taken into account that naming abilities vary according to the semantic feature of the object, as well as its familiarity.^6^

Verbs are considered to be more complex to name, as they present greater semantic and grammatical variety, and are more difficult to identify according to their classifications as action verbs (e.g. “pull”), process verbs (e.g. “happen”), action-process (e.g. pronoun followed by the infinitive of the verb), state (e.g. “want”) and auxiliary (such as an auxiliary verb followed by an infinitive).^22^

Another factor that increases verb complexity is their syntactical analysis, by which verbs are divided into transitive and intransitive^21^. Transitive verbs can also be subdivided into Direct Transitive – i.e., those which transit directly to the complement without the need for a preposition (as in “I heard the noise”), Indirect Transitive – i.e., those which transit to the complement and take a preposition (as in “I believe in ghosts”), and Direct and Indirect Transitive, i.e. are bound to the complement directly and indirectly (as in “I wrote a letter to the President”).

Intransitive verbs, on the other hand, have a complete meaning and therefore do not need a complement (as in “The butterfly died”).

Luzzatti^18^ reported that in aphasic subjects the process of naming of transitive verbs is more compromised than for intransitive verbs because the transitive verbs have greater syntactic complexity due to the use of verbal complements.

## Neural substrate of linguistic processing of verbs and objects

Neurofunctional studies of cortical activation using magnetic resonance (fMRI) in the production of verbs and objects have been conducted in order to determine the precise brain regions involved in these abilities.

Studies in brain-injured subjects suggest that verb production is related to the left frontal cortex, including regions of the ventrolateral prefrontal cortex, posterior frontal gyrus, and Broca’s area.^20,24,25^

The perisylvian area has also been studied and has been shown to be connected to the motor and premotor cortices through a subcortical pathway including the basal ganglia and the anterior portion of the left thalamus.^18^

The production of nouns, on the other hand, is related to the temporal lobes bilaterally: the mid portion of the left fusiform gyrus and the mid portion of the right superior temporal gyrus.^24,26,27^

Damasio^28^ stated that subjects with left temporal lesions present disturbances in the production of proper names, while right temporal lesions compromise their recognition. When the lesion extends to the anterior portion of the left inferior temporal cortex, the subject has impaired production of non living names, but the recognition of stimuli will be affected if the lesion involves the right mesial temporo-occipital cortex.

## Naming processes in aphasia

The classical aphasia classification (Wernicke-Geschwind’s) is based on the subject’s performance in several aspects of language, such as spontaneous speech (fluency), comprehension, and repetition.^29^

Aphasia can be also divided into two main groups: fluent and non fluent.^9,29^ According to the aforementioned classification, the fluent aphasias are Anomic aphasia, Conduction aphasia, Wernicke’s aphasia, and Sensory Transcortical aphasia. They are characterized by normal, or sometimes augmented verbal production (logorrhea), paraphasias, and jargonaphasia, with varying degrees of comprehension impairment. Non fluent aphasias comprise Broca’s aphasia, Motor Transcortical aphasia and Global aphasia, which are characterized by effortful production, articulatory slowing, aprosodia, reduced sentence length, and dysarthria.

A common characteristic in all types of aphasia is the presence of anomia.^7,8^ According to Penã-Casanova,^4^ anomia, or lexical processing impairment, can be used to distinguish the various clinical forms of aphasia.

In Broca’s aphasia, there is impairment in spontaneous speech, with perseverations and agrammatism. In Wernicke’s aphasia, verbal paraphasias and neologisms are frequent, both in spontaneous speech and in formal tests of oral naming. In Sensory Transcortical aphasia, semantic paraphasias and circumlocutions occur, while in Motor Transcortical aphasia, there is an increasing latency on naming tasks with frequent perseverations. In Conduction aphasia, phonemic paraphasias predominate. In some cases, anomia is the only language alteration patients, and this type of Anomic aphasia may either be primary or residual (evolving from other forms of aphasia).

Recently, several studies have sought to investigate the neuroplasticity and neural reorganization involved in the recovery of anomic patients, with or without specific rehabilitation measures.

Cornelissen^30^ described three chronically aphasic subjects with left hemisphere lesions, for whom an intensive rehabilitation method targeting their lexical access difficulties was proposed, including activities with repetition priming and semantic priming. The authors evidenced an improvement in left inferior parietal cortical activity, suggesting that this modality of training induces an improvement in the phonological buffer span of verbal working memory (phonological loop).

Conroy^31^ studied seven aphasic subjects, with different aphasia types and severity, using ten sessions of verb and noun naming therapy, in which subjects had to name isolated pictures, and pictures related to narratives. The results of the therapy suggested that an increase in the precision when naming isolated pictures may lead to an improvement in contextualized naming (i.e. in discourse).

Ortiz^32^ compared the performance of thirty aphasic subjects and thirty controls matched for schooling, on a verb and noun naming task. Among the aphasics, those with five or more years of schooling performed better in the naming of actions.

Laganaro^33^ reported a case of a subject with severe anomic aphasia, who had been enrolled as a control in a previous study with anomic aphasia. The comparison of data pre and post left hemisphere stroke corroborated the role of the left temporal cortex in semantic-phonological processing of naming tasks.

## Discussion

We found that current studies investigating naming abilities tended to emphasize the differentiation between nouns and verbs both in terms of their lexical properties and neuroanatomical substrates.

The left inferior temporal cortex is described as related to object naming, while the visual recognition of objects is predominantly related to the right temporo-occipital cortex.^28^ By contrast, motor and premotor areas in the left frontal cortex are related to the production of verbs.

The combination of clinical evaluation and functional neuroimaging has contributed greatly to a better understanding of the neural networks involved in naming processes, and has helped highlight the differences between words that have diverse linguistic properties, such as nouns and verbs.

Naming abilities are relevant in the context of Neurolinguistic studies as they still represent the gold standard parameter of lexical access. This holds for both formal tests as well as broader tasks such as discourse production, in which the subject has to maintain activation of the neural networks that allow constant lexical access, with semantic and grammatical modulations, constituting the basis of spontaneous speech.^4^

To summarize, clinico-functional studies have improved our knowledge on category-specific naming abilities. However, in order to optimize rehabilitation efforts and increase their efficacy, further studies involving different types of aphasia and naming abilities in different contexts (especially discourse and spontaneous speech) are needed. Comparisons among different kinds of intervention and their respective impact on the recovery of anomia would also be valuable.

Finally, studies in this particular field of Neurolinguistics remain scarce in Brazilian Portuguese, indicating an open field to be explored, considering the grammatical specificities of our language, both regarding noun (for example: our derivation processes in augmentative and diminutive forms) and verb conjugation rules.

Cross-cultural studies comparing Brazilian Portuguese to other languages would also be of great interest for detecting the similarities and differences in the neural organization of category-specific naming processing.
